# The Functional Interaction between Acyl-CoA Synthetase 4, 5-Lipooxygenase and Cyclooxygenase-2 Controls Tumor Growth: A Novel Therapeutic Target

**DOI:** 10.1371/journal.pone.0040794

**Published:** 2012-07-13

**Authors:** Ulises D. Orlando, Juan Garona, Giselle V. Ripoll, Paula M. Maloberti, Ángela R. Solano, Alejandra Avagnina, Daniel E. Gomez, Daniel F. Alonso, Ernesto J. Podestá

**Affiliations:** 1 Instituto de Investigaciones Biomédicas (INBIOMED), Department of Human Biochemistry, School of Medicine, University of Buenos Aires, Buenos Aires, Argentina; 2 Laboratory of Molecular Oncology, Quilmes National University, Buenos Aires, Argentina; 3 Pathology Unit, Hospital Universitario-Centro de Estudios Médicos e Investigación Clínica (CEMIC), Buenos Aires, Argentina; Wayne State University School of Medicine, United States of America

## Abstract

The acyl-CoA synthetase 4 (ACSL4), which esterify mainly arachidonic acid (AA) into acyl-CoA, is increased in breast, colon and hepatocellular carcinoma. The transfection of MCF-7 cells with ACSL4 cDNA transforms the cells into a highly aggressive phenotype and controls both lipooxygenase-5 (LOX-5) and cyclooxygenase-2 (COX-2) metabolism of AA, suggesting a causal role of ACSL4 in tumorigenesis. We hypothesized that ACSL4, LOX-5 and COX-2 may constitute potential therapeutic targets for the control of tumor growth. Therefore, the aim of this study was to use a tetracycline Tet-Off system of MCF-7 xenograft model of breast cancer to confirm the effect of ACSL4 overexpression on tumor growth *in vivo*. We also aim to determine whether a combinatorial inhibition of the ACSL4-LOX-COX-2 pathway affects tumor growth *in vivo* using a xenograft model based on MDA-MB-231 cells, a highly aggressive breast cancer cell line naturally overexpressing ACSL4. The first novel finding is that stable transfection of MCF-7 cells with ACSL4 using the tetracycline Tet-Off system of MCF-7 cells resulted in development of growing tumors when injected into nude mice. Tumor xenograft development measured in animals that received doxycycline resulted in tumor growth inhibition. The tumors presented marked nuclear polymorphism, high mitotic index and low expression of estrogen and progesterone receptor. These results demonstrate the transformational capacity of ACSL4 overexpression. We examined the effect of a combination of inhibitors of ACSL4, LOX-5 and COX-2 on MDA-MB-231 tumor xenografts. This treatment markedly reduced tumor growth in doses of these inhibitors that were otherwise ineffective when used alone, indicating a synergistic effect of the compounds. Our results suggest that these enzymes interact functionally and form an integrated system that operates in a concerted manner to regulate tumor growth and consequently may be potential therapeutic targets for the control of proliferation as well as metastatic potential of cancer cells.

## Introduction

Breast cancer is the most frequent malignant disease in women and the second leading cause of cancer-related deaths in the U.S., affecting one in eight Americans throughout their lifetime [1]. Mechanisms involved in the frequent failure of chemotherapy, endocrine therapy or immunotherapy to successfully treat breast cancer are elusive and are being investigated. Breast cancer cells in a patient are heterogeneous, differing in their manifest state of differentiation and malignant potential [2].

Random mutation events and/or epigenetic changes of cancer cells followed by the selection of more malignant variants or the acquisition of stem cell-like properties are thought to be the mechanism for tumor progression and consequently for the generation of a heterogeneous tumor cell population [3], [4].

Cancer is a disease with genomic perturbation that leads to dysregulation of multiple pathways within the cellular system. Of these pathways, alterations in arachidonic acid (AA) metabolism have been suggested to contribute to tumorigenesis and tumor progression [5], [6], [7], [8]. Yet, the direct impact of this knowledge on tumor treatment and prevention is still largely unproven.

Increased expression of enzymes involved in AA metabolism, cyclooxigenase-2 (COX-2) and lipooxigenase-5 (5-LOX), has been reported in aggressive metastatic breast cancer cells [9], [10]. A number of studies have used chemically-induced mammary carcinogenesis models or other models having endogenously high levels of COX-2 to demonstrate a role for COX-2 and prostaglandin E_2_ (PGE_2_) in mammary tumors [11], [12], [13]. These models have significantly advanced our knowledge of the central role played by of COX-2 and PGE_2_ in mammary tumor development, in resistance to apoptosis, as well as of the role of PGE_2_ in the “angiogenic switch” that activates development of new blood vessels, considered essential for tumor expansion and invasion [13], [14], [15]. The models described above have also been useful to study the growth rate of various solid tumors following administration of COX-2 inhibitors [14].

The potential therapeutic benefit of COX-2 inhibitors in a range of cancers is being seen as a great promise; however, since recent concerns about potential cardiotoxicity [16], [17] has generated an urgency to develop new inhibitors with a better risk/benefit ratio.

Abnormal expression of acyl-CoA synthetase-4 (ACSL4) has been documented in colon adenocarcinoma, hepatocellular carcinoma and breast cancer [18], [19], [20], [21]. ACSL4 belongs to a five-member family of enzymes that esterify mainly AA into acyl-CoA [22], [23]. We previously demonstrated that the sole transfection of MCF-7 cells, a model of non-aggressive breast cancer cells, with ACSL4 cDNA, transforms those cells into a highly aggressive phenotype [21]. We found that levels of LOX and COX-2 products of AA are regulated by ACSL4 expression in a breast cancer cell line. Functionally, we found that ACSL4 is part of the mechanism responsible for increased breast cancer cell proliferation, invasion and migration [21]. Based on our results, we hypothesized that ACSL4, LOX-5 and COX-2 may constitute potential therapeutic targets for the control of tumor growth. Therefore, the aim of this study was to use a tetracycline Tet-Off system of MCF-7 xenograft model of breast cancer to demonstrate the effect of ACSL4 overexpression on tumor growth *in vivo*. We also aimed to determine whether a combinatorial inhibition of the ACSL4-LOX-COX-2 pathway affects tumor growth *in vivo* on MDA-MB-231 tumor xenografts.

## Results

We previously demonstrated that overexpression of ACSL4 results in a significant increase in the proliferation and invasion rate of MCF-7 cells compared to mock-transfected cells [21]. Our results were confirmed by stable transfection of MCF-7 cells with ACSL4 using the tetracycline Tet-Off system [21].

To investigate whether the sole overexpression of ACSL4 could transform MCF-7 cells into an *in vivo* tumor-forming phenotype, our strategy was therefore based on injecting mice with cells endowed with the capacity to form tumors and to reduce or prevent tumor formation by controlling ACSL4 expression. The evaluation of tumor growth and its consequent reduction by a known variable is more reliable than evaluating the lack of tumor formation by modulating a “normally” tumor-forming cell by manipulating the cells.

### *In vivo* Development of MCF-7 Tet-Off/ACSL4 Tumor Xenografts

**Figure 1:**
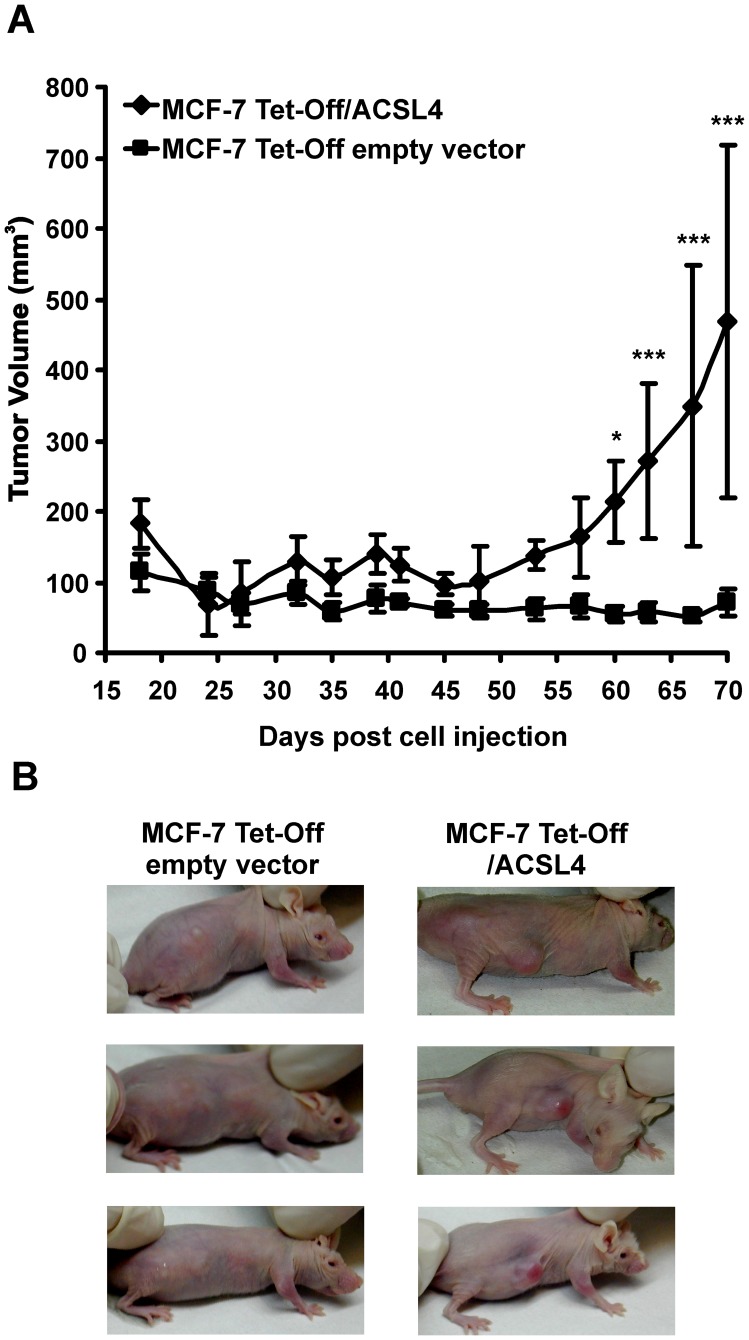
*In vivo* development of MCF-7 Tet-Off/ACSL4 tumor xenografts. (A) Comparison of average tumor volume of MCF-7 Tet-Off/ACSL4 and MCF-7 Tet-Off empty vector tumor xenografts 70 days after injection of 5×10^6^ cells into the right flank of female Balb/c nu/nu mice, aged 6–8 weeks. Each point represents mean ± SD, n = 5. GraphPad Prism Software was used to performed a two-way ANOVA, post test Bonferroni. (*) *P*<0.05; (***) *P*<0.001. (B) A representative photograph of mice carrying MCF-7 Tet-Off/ACSL4 and MCF-7 Tet-Off empty vector tumors.

In contrast with the highly aggressive MDA-MB-231 cells which develop into mammary tumors when transplanted into animals [24], inoculation of MCF-7 cells into 6–8 week-old female Foxn1 nu/nu Balb/c athymic nude mice without exogenously added estrogen did not form growimg tumors (Figure 1A). In contrast, inoculation of female athymic mice with the stable cell line MCF-7 Tet-Off/ACSL4 resulted in the development of mammary tumors (Figure 1A), thereby demonstrating the transformational capacity of MCF-7 Tet-Off/ACSL4 cells. Palpable tumors developed 15 days after cell inoculation. Seventy days after injection, tumors were significantly larger in animals inoculated with MCF-7 Tet-Off/ACSL4 cells compared to the mass observed when animals were inoculated with MCF-7 Tet-Off empty vector cells analyzed by two-way ANOVA (Figure 1A). Tumor volumes obtained by inoculation of MCF-7 Tet-Off/ACSL4 cells reached values similar to those described when animals were inoculated with the highly aggressive MDA-MB-231 cell line [24], although the time required to reach that size was twice as long (70 vs 35 days, respectively). Representative tumor samples from each treatment group are shown in Figure 1B.

Thus far, our results show that the sole transfection of ACSL4 results in a phenotype change that endows cells with the capacity to develop into tumors when injected into nude mice.

**Figure 2:**
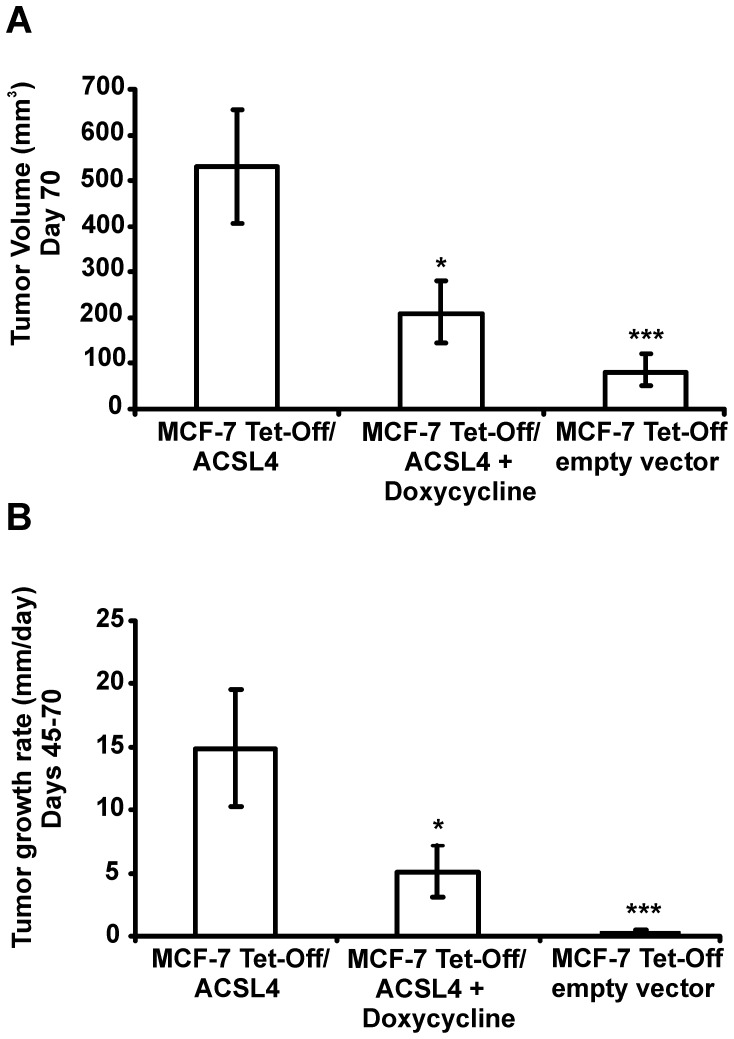
Tumor xenograft development measured in animals that received doxycycline. Comparison of average tumor volume (A) and tumor growth rate (B) of MCF-7 Tet-Off/ACSL4 tumor xenografts after injection of 5×10^6^ cells into the right flank of female Balb/c nu/nu mice, aged 6–8 weeks treated with or without doxycycline and average tumor volume of MCF-7 Tet-Off empty vector tumor xenografts 70 days after injection of 5×10^6^ cells into the right flank of female Balb/c nu/nu mice, aged 6–8 weeks. Values are mean ± SD, n = 5. The asterisk indicates significant differences between the tumor volumes and rate of tumor growth by one-way ANOVA post test Student-Newman-Keuls (*) *P*≤0.05 and (***) *P*≤0.001.

Tetracycline-controlled transcriptional activation (Tet-On/Off) is a method of inducible expression in which transcription is reversibly turned either on or off in the presence of the antibiotic tetracycline or doxycycline. To further assess the role of ACSL4 in the tumorigenic capacity of MCF-7 cells, nude mice were inoculated s.c. with MCF-7 Tet-Off/ACSL4 cells as described in Figure 1 and tumor xenograft development was measured in animals that received doxycycline (2 mg/ml) or vehicle in drinking water. As expected, the, treatment of nude mice with doxycycline resulted in tumor growth inhibition (Figure 2A). The tumor growth rate of MCF-7 Tet-Off/ACSL4 tumor xenografts between days 45 and 70 was significantly higher than that in doxycycline-treated animals (Figure 2B) and than that in animals inoculated by inoculation with MCF-7 Tet-Off empty vector cells.

### Histopathology of MCF-7 Tet-Off/ACSL4 Tumor Xenografts

**Figure 3:**
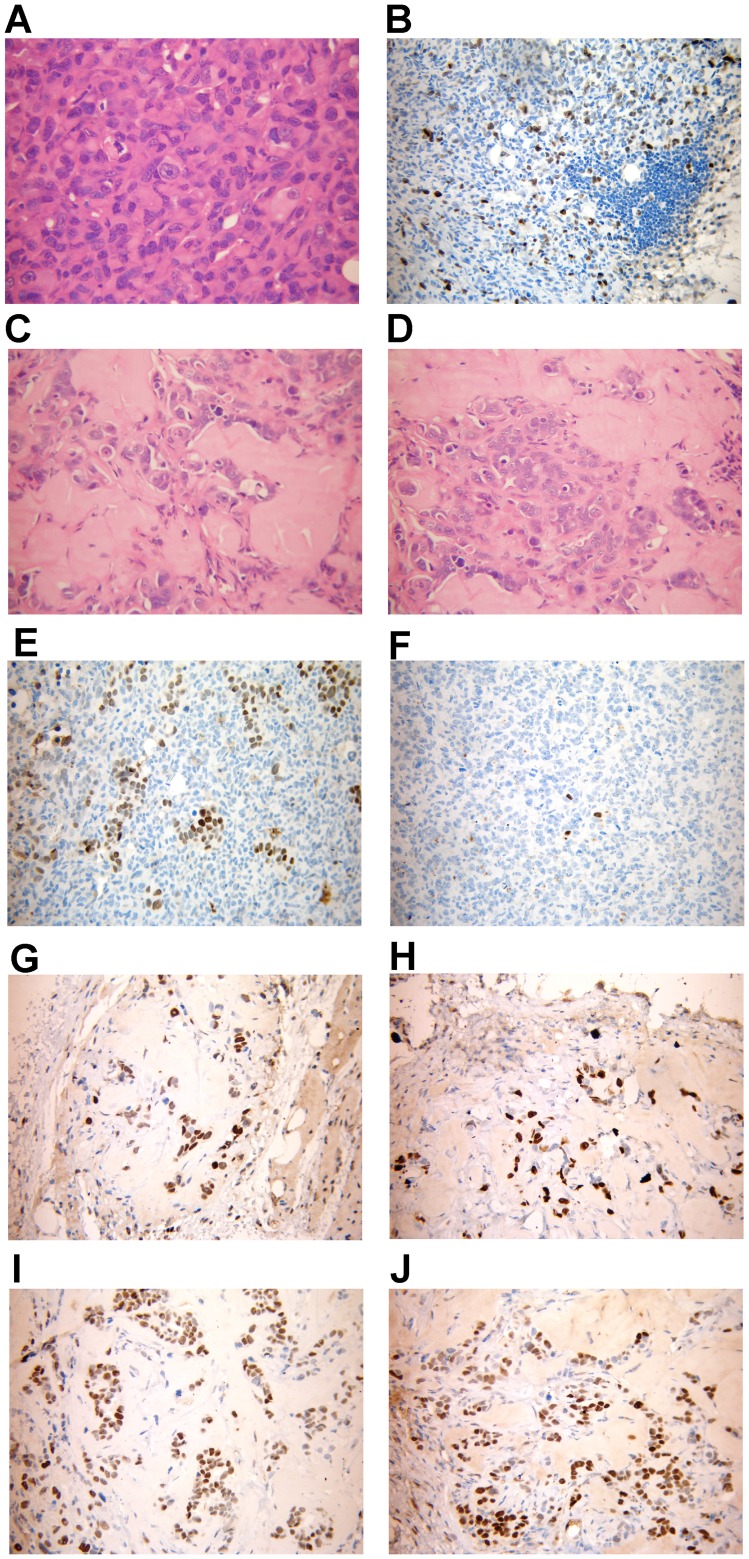
Histopathology and Immunohistochemical analysis of MCF-7 Tet-Off/ACSL4 tumor xenografts. Histological analysis of human breast tumors formed 70 days after injection of 5×10^6^ MCF-7 Tet-Off/ACSL4 cells into the right flank of female Balb/c nu/nu mice, aged 6–8 weeks. Panel A, C and D showed a representative hematoxylin & eosin stained tissue sections of MCF-7 Tet-Off/ACSL4, MCF-7 Tet-Off empty vector, and MCF-7 Tet-Off/ACSL4 plus doxycyclin treated tumors respectively. Panel B showed a representative immunohistochemical analysis of Ki-67 stained of MCF-7 Tet-Off/ACSL4 tumors. Tumor specimens were stained for detection of ERα and PR receptor expression using the specific antibodies as described in Materials and Methods. Panels showed a representative inmunohistochemical analysis of ERα and PR of the tumor from MCF-7 Tet-Off/ACSL4 xenografts (panel 3E and 3F respectively); from MCF-7 Tet-Off empty vector (panel 3G and 3H respectively) and from MCF-7 Tet-Off/ACSL4 xenografts treted with doxycycline (panel 3I and 3J respectively.

The MCF-7 Tet-Off/ACSL4 tumor xenografts, the MCF-7 Tet-Off empty vector tumor xenografts, and the MCF-7 Tet-Off/ACSL4 tumor xenografts treated with doxicycline (Figure 3) were isolated, stained with hematoxylin–eosin and analyzed under light microscopy in order to define the histology of the tumors (Figure 3).

The histological grade of the MCF-7 Tet-Off/ACSL4 tumor xenografts was evaluated by the Elston/Nottingham criteria [25]. The tumors presented marked nuclear polymorphism and displayed very high cell density (Figure 3A). The number of mitotic figures in the most active area determined by a count of 10 high power fields revealed that the tumors were poorly differentiated (grade II/III). The tumor also presented some lymphocyte infiltrate. MCF-7 Tet-Off empty vector cells produced tumors of negligible size, and we identified very small tumors in only two animals out of ten that were inoculated with the MCF-7 Tet-Off empty vector cells. The tumor from MCF-7 Tet-Off empty vector cells presented areas of hyaline strome with few tumor cells and few glands and was classified as a differentiated tumor (Figure 3 C).The MCF-7 Tet-Off/ACSL4 tumor xenografts treated with doxycycline also presented areas oh hyaline strome with few tumor cells and was classified as differentiated tumor (Figure 3 D).

Immunohistochemical analysis of the MCF-7 Tet-Off/ACSL4 tumor xenografts with the Ki-67 antibody revealed increased proliferation of ACSL4 overexpressing tumors with a high mitotic index as demonstrated by Ki-67 staining (Figure 3 B).

Using immunocytochemical analysis the MCF-7 human breast cancer cell line has been classified as a breast carcinoma luminal subtype expressing the estrogen receptor (ER) and progesterone receptor (PR) without HER2 overexpression and moderate levels of Ki-67 [26]. In contrast, the MDA-MB-231 human breast cancer cell has been classified as a basal-like subtype negative for ER, PR and HER2 with high levels of Ki-67 [26].

**Figure 4:**
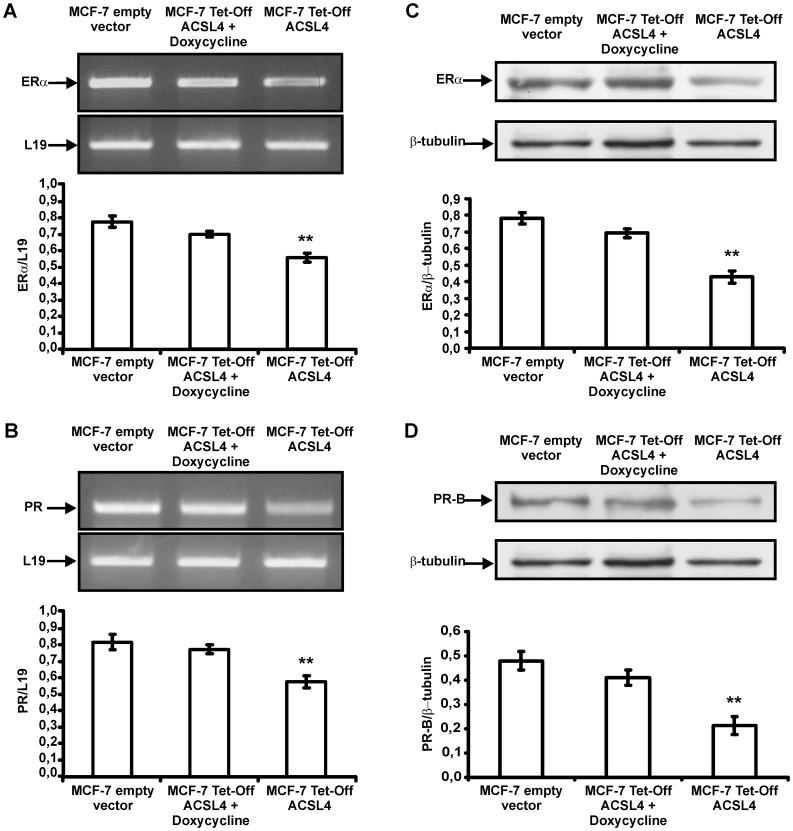
ERα and PR receptor expression in MCF-7 Tet-Off empty vector and MCF-7 Tet-Off/ACSL4 human breast cancer cells. MCF-7 Tet-Off/ACSL4 cells were incubated with or without docicycline. After 96 h of doxycycline treatment, total RNA and cellular proteins from MCF-7 Tet-Off empty vector and MCF-7 Tet-Off/ACSL4 cells were extracted and subjected to RT-PCR analysis for ERα and PR and L19 mRNA expression and SDS-PAGE and Western blot analysis respectively. Semiquantitive RT-PCR of ERα and PR (panel A and B respectively). were performed as described in Materials and Methods. The integrated optical density of PCR products was normalized with the corresponding L19 mRNA bands. Data represent mean ± SD of three independent experiments. Western blot analysis of ERα and PR (panel C and D respectively) were performed as described in Materials and Methods. Using anti ERα and PR - and anti-β-tubulin antibodies, specific protein bands were detected in immunoblots by enhanced chemiluminescence. The integrated optical density of protein levels was quantified and normalized with the corresponding -tubulin signal. Data represent the means ± SD of three independent experiments. The asterisk indicates significant differences analyzed by Student’s test comparing MCF-7 Tet-Off empty vector and MCF-7 Tet-Off/ACSL4 cells, (**) *P*≤0.05.

The MCF-7 Tet-Off/ACSL4 human breast cancer cells showed a significant reduction in ERα and PR, mRNA and protein expression (Figure 4 A and C, B and D respectively). Treatment of the MCF-7 Tet-Off/ACSL4 human breast cancer cells with doxycycline for 96 h return the levels of ERα and PR mRNA and protein expression to the levels of MCF-7 Tet-Off empty vector (control cells).

Inmunohistochemical analysis of the tumor from animal inoculated with MCF-7 Tet-Off/ACSL4 breast cancer cell showed few positive cells expressing ERα (Figure 3 E) and a very few stained for PR (Figure 3 F). The expression of HER2 was negative in MCF-7 Tet-Off/ACSL4 tumor xenografts (data not shown). Further, when a 4-mm^3^ fragment from surgically-resected MCF-7 Tet-Off/ACSL4 tumors was transplanted into acceptor female nude mice a new tumor measuring 2,500 mm^3^ developed 49 days after inoculation. This procedure was repeated and following this third passage a new tumor of 5,000 mm^3^ and a metastatic node developed 50 days after inoculation.

Inmunohistochemical analysis of the tumor from animals inoculated with MCF-7 Tet-Off empty vector and from from animal inoculated with MCF-7 Tet-Off/ACSL4 and treated with doxycycline showed almost all cells expressing ERα (figure 3 G and H) and PR (figure 3 I and J) respectively. These results suggest that the expression of ACSL4 negatively controlled the expression of ERα and PR during the tumor growth.

These data demonstrate a significant role for ACSL4 in tumor proliferation, survival and progression. To our knowledge, this is the first study to focus solely on ACSL4 overexpression and its biological impact on breast cancer *in vivo*.

Overexpression of ACSL4 regulates COX-2 expression through out the increase in LOX-5 activity [21]. Thus this mechanism could be used to study the potential action of a combination of inhibitors of COX-2 regulation and function. Therefore, we proceeded to study the effect of a combination of inhibitors of the different enzymes in an *in vivo* model.

### Treatment with Inhibitors of COX-2, LOX-5 and ACSL4 Effectively Limited Tumor Growth of Human MDA-MB-231 Breast Cancer Xenografts

Thus far, our previous in vitro results and the demonstration that modulating ACSL4 expression results in the up-regulation of COX-2 and LOX activities with a consequent change in cell phenotype.

The logical next step was to analyze the effect of ACSL4, COX-2 and LOX inhibitors on tumor growth in vivo. The MDA-MB-231 cell line is known to naturally overexpress the three enzymes, to form tumors with a triple-negative signature that do not respond to hormone treatment and are very resistant to radio o chemotherapy. This has led to a widespread use of the MDA-MB-231 xenograft model to study the efficacy of different treatments. The MDA-MB-231 is therefore the natural model to investigate if a combinatorial therapy targeting ACSL4, COX and LOX is effective in reducing tumor growth. Therefore, the MDA-MB-231 xenograft model was a very good challenge to demonstrate that the enzymes are working in a concerted manner and to also demonstrate a synergistic effect of the inhibitors as a potential therapeutic protocol.

While the Tet-Off/ACSL4 xenograft model generated with MCF-7 cells is a useful tool to demonstrate the role of ACSL4 in breast cancer pathogenesis, the MDA-MB-231 confirms the natural occurrence of this mechanism is therefore the proper model to investigate that the proposed mechanism could be constitute a possible therapeutic target.

A colon cancer cell model demonstrated that pharmacological inhibitors of ACSL4 and COX-2 show an additive effect in reducing cell proliferation [27]. In previous studies, we have observed the same additive and inhibitory effect of a combination of ACSL4 and COX-2 inhibitors on cell proliferation of MDA-MB-231 breast cancer cells (unpublished data). Surprisingly, when we used pharmacological inhibitors of ACSL4, LOX-5 and COX-2 to determine whether they could inhibit cell proliferation and migration *in vitro*, we observed a synergistic effect of these inhibitors.

MDA-MB-231 breast cancer cells were treated with all possible combinations of two different inhibitors of ACSL4, LOX-5 and COX-2. The minimal doses that exerted a significant inhibitory effect were: 75 µM for rosiglitazone and troglitazone; 10 and 500 µM for AA861 and zileuton and 1 and 500 µM for etoricoxib and ibuprofen, for ACSL4, LOX-5 and COX-2 respectively. Based on these results we use the inhibitors in doses that are ineffective by themselves (Table 1). The results show that none of the employed combinations of two different inhibitors of ACSL4, LOX-5 and COX-2 produced a synergistic inhibition on cell proliferation and migration of MDA-MB-231 breast cancer cells (Table 1). In contrast, the triple therapy was the only combination that produced a synergistic inhibitory effect on cell proliferation or migration of MDA-MB-231 cells (Table 1). The same results were obtained using the HS578T breast cancer cell line (Table 2).

**Table 1:** Effect of enzyme inhibitors on cell proliferation and migration of MDA-MB-231 cells.

Treatment	Cell proliferation (% of control)	Wound healing (% of control)
Control	100	100
Trog/Ibu	95,8±5,2	97,3±6,7
Trog/Eto	96,2±5,5	98,1±5,2
Trog/Zil	95,4±5,8	98,4±6,1
Trog/AA861	96,8±6,0	96,2±5,3
Ros/Ibu	99,3±4,1	98,2±6,8
Ros/Eto	98,4±3,8	98,5±4,2
Ros/Zil	98,2±5,0	97,9±5,4
Ros/AA861	97,6±5,3	96,4±6,5
Ibu/Zil	99,8±3,3	97,8±5,4
Ibu/AA861	97,4±4,3	94,8±6,3
Eto/Zil	96,4±5,3	95,6±5,2
Eto/AA861	97,3±4,8	96,1±4,8
TriC/Ibu	96,8±5,2	98,6±5,1
TriC/Eto	97,1±4,8	97,4±6,3
TriC/Zil	96,4±4,1	98,1±5,9
TriC/AA861	95,3±4,0	97,3±5,0
Trog/Ibu/Zil	59,4±6,8 **	73,4±3,9 *
Trog/Eto/Zil	63,8±5,9**	70,4±4,8 *
Trog/Ibu/AA861	65,4±6,3*	68,9±4,9 *
Trog/Eto/AA861	59,9±5,2**	64,3±5,2 **
Ros/Ibu/Zil	62,4±3,8 **	73,4±3,8 *
Ros/Eto/Zil	60,3±2,9 **	71,0±3,4 *
Ros/Ibu/AA861	58,4±3,8 **	70,3±4,8 *
Ros/Eto/AA861	57,4±2,9 **	67,4±6,1 *
TriC/Ibu/Zil	53,4±2,3 **	68,3±5,6 *
TriC/Eto/Zil	50,4±2,6 **	65,4±6,5 *
TriC/Ibu/AA861	56,4±2,8 **	67,6±6,0 *
TriC/Eto/AA861	50,3±3,0 **	65,9±4,8 *

MDA-MB-231 tumor cells treated with either vehicle or roziglitazone (Ros), troglitazone (Trog), Triasin C (TriC), zileuton (Zil), AA861, ibuprofen (Ibu), etoricoxib (Eto) as ACSL4, LOX-5 and COX-2 inhibitors, respectively, alone or in combination as indicated were incubated during 12 or 96 h and cell migration and proliferation assayed as described in Materials and Methods.Doses employed were: 25 µM for Ros and Trog; 100 µM and 5 µM for Zil and AA861 respectively; 100 µM and 0.01 µM for Ibu and Eto respectively and 5 µM for TriC.

Data represent the mean ± SD of 2 or 3 independent experiments for cell migration and proliferation, respectively, performed in quintuplicate. * P<0.05 and ** P<0.01 vs. Control.

**Table 2:** Effect of enzyme inhibitors on cell proliferation and migration of HS-578-Tcells.

Treatment	Cell proliferation (% of control)	wound healing (% of control)
Control	100	100
Ros/Ibu	95,4±5,8	97,1±6,8
Ros/Zil	97,3±4,6	94,1±5,9
Ibu/Zil	96,2±5,1	97,4±6,1
Ros/Ibu/Zil	57,1±5,2 **	68,3±5,8 *
Tric/Ibu	96,2±6,8	97,3±6,0
Tric/Zil	97,4±5,8	96,8±7,1
Tric/Ibu/Zil	55,3±6,1 **	65,3±5,0 *

Cell migration and proliferation were determined as described in table 1. Data represent the mean ± SD of 2 or 3 independent experiments for cell migration and proliferation, respectively, performed in quintuplicate. * P<0.05 and ** P<0.01 vs. Control.

Based on these results, we hypothesized that ACSL4, COX-2 and LOX-5 could be potential therapeutic targets for the control of tumor growth and that the use of a combination of inhibitors would result in potentiation of their effect compared to therapy with a single-drug. A possible synergistic effect of combined therapy could result in increased efficacy and/or reduced deleterious effects. Therefore, to broaden the potential clinical applications of this finding and to address whether our results obtained in cancer cell lines are relevant for tumor growth *in vivo*, we tested a possible synergistic effect of a combination of inhibitors on breast cancer tumor growth in an *in vivo* model.

For that purpose, we used a xenograft model with which we examined the effect of inhibitors of ACSL4, LOX-5 and COX-2 on tumors formed after injection of MDA-MB-231 human breast cancer cells into nude mice.

Based on the pharmacokinetic profile of the drugs, which exhibit a similar elimination half life, the combination therapy used was: rosiglitazone as ACSL4 inhibitor [28], zileuton as LOX-5 inhibitor [29], [30] and ibuprofen as a non-selective COX-2 inhibitor [31], [32]. We assayed the effectiveness of therapy based on a combination of sub-effective doses of the different inhibitors.

Following a 4-day window to allow the establishment of tumor xenografts in mice injected with tumor cells, the mice were randomly separated into groups and daily treated intraperitoneally (i.p.) with the inhibitors or appropriate negative controls. Treated mice were killed on day 30 post tumor cell injection and tumor samples were collected.

**Figure 5:**
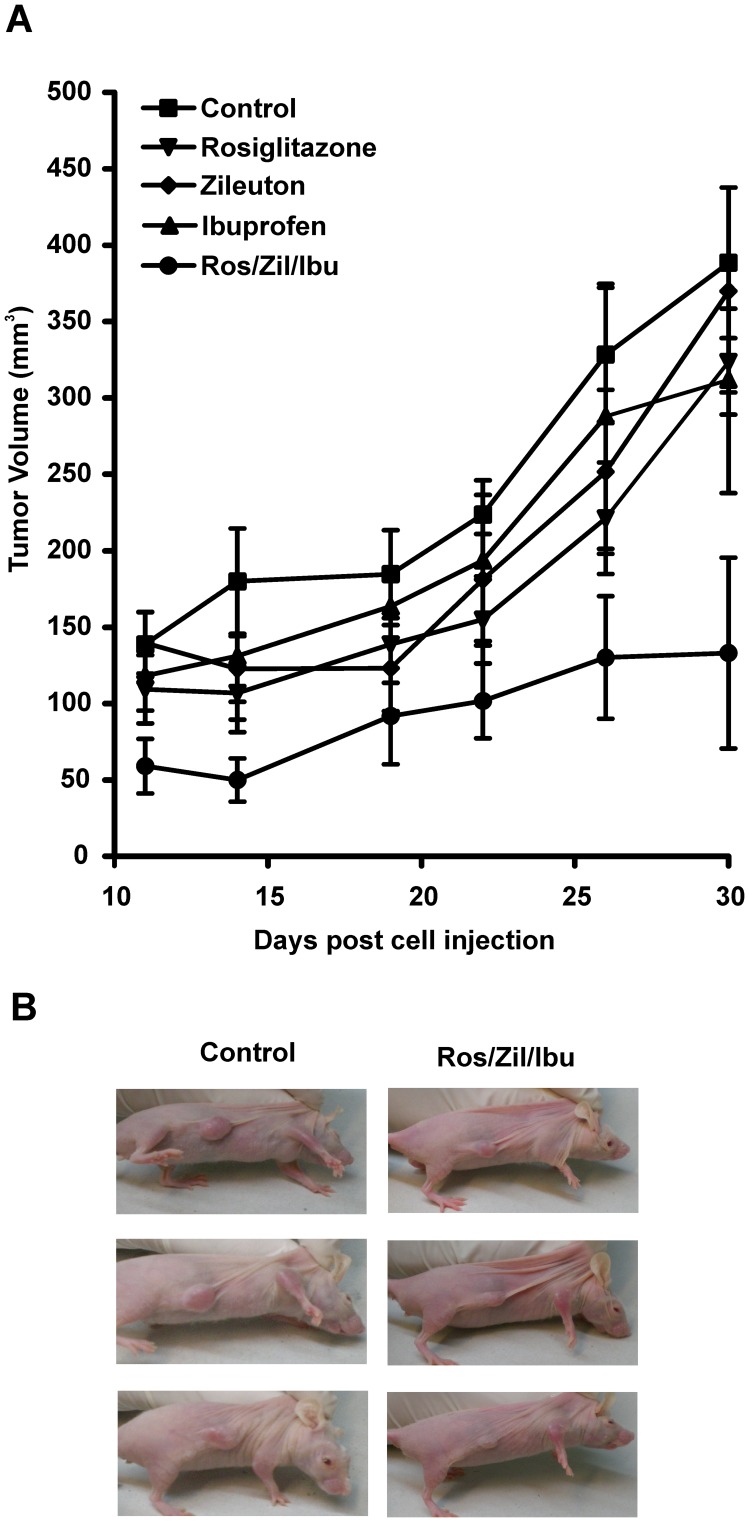
Intraperitoneal administration of inhibitors in the MDA-MB-231 human breast xenograft model. A) Tumor growth inhibition curve in female athymic nude mice bearing MDA-MB-231 tumor xenografts treated intraperitoneally with either vehicle or roziglitazone, or zileuton, or ibuprofen alone or a combination of the three inhibitors (Ros/Zil/Ibu) at doses described in Materials and Methods for twenty-six consecutive days beginning on day 4 post implantation of 5×10^6^ MDA-MB-231 human breast cancer cells. Each point represents mean ± SD, n = 5. B) A representative photograph of mice bearing MDA-MB-231 tumor xenograft.

**Figure 6:**
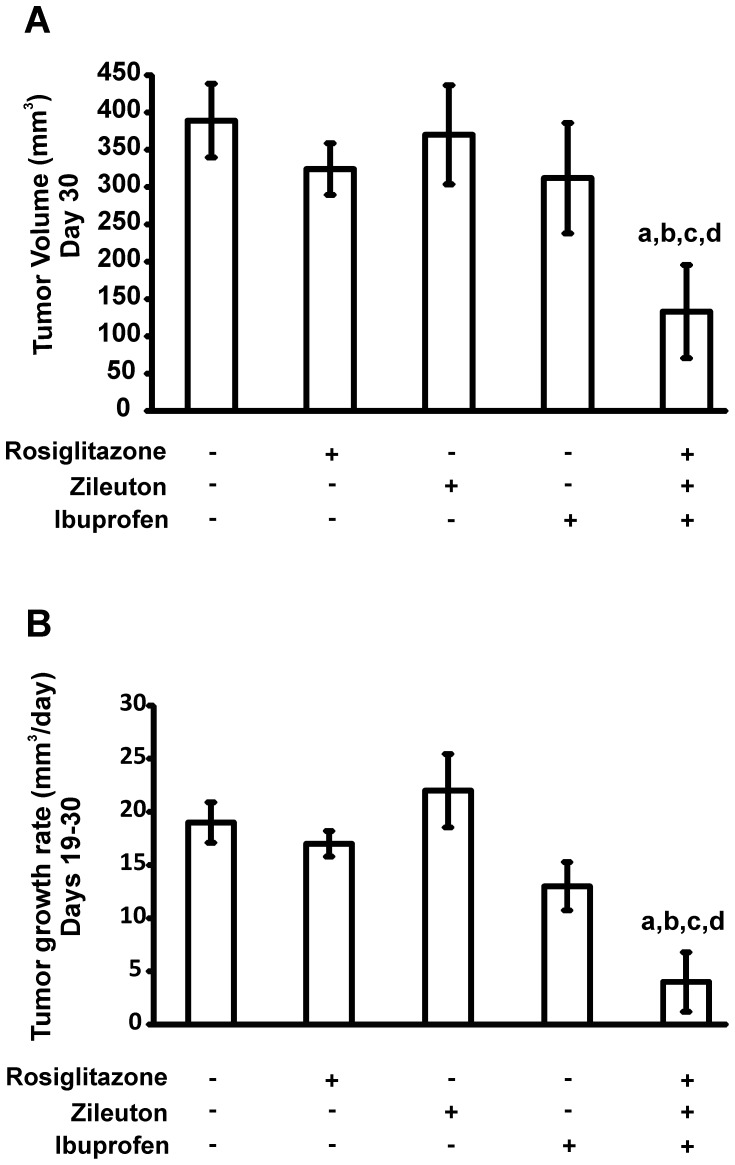
Effect of enzyme inhibitors on average tumor volume and tumor growth rate of MDA-MB-231 tumor xenografts. Comparison of average tumor volume (A) and tumor growth rate (B) of MDA-MB-231 tumor xenografts treated with either vehicle or roziglitazone, or zileuton, or ibuprofen alone or a combination of the three inhibitors at doses described in Materials and Methods. Values are mean ± SD, n = 5. The characters indicate significant differences between tumor volume and rate of tumor growth by one-way ANOVA, post test Bonferroni. a, *P*<0.001 vs vehicle; b, *P*<0.001 vs rosiglitazone; c, *P*<0.001 vs zileuton; d, *P*<0.01 vs ibuprofen.

Although the MDA-MB-231 xenograft growth rate varies among studies reported in the literature, our tumor xenografts were in the range of those reported previously [24]. The average animal body weight was 23.5 g at the beginning of the treatment and no significant differences in body weight were observed between the different treatment groups at the end of the experiment. The amount of food intake in the control compared to the treated groups was not significantly different throughout the experiment. However, as shown in Figure 5A there was a significant inhibition in tumor growth subjected to combination therapy compared to those that received single drug-based treatments or drug vehicle after injection of MDA-MB-231 cells. A two-way ANOVA test was used to analyze the data. Both variables under study, days post-injection and treatment with the different inhibitors, as well as the interaction between the two variables produced a highly significant (P<0.001) effect on tumor growth. The results of a post-hoc test (Bonferroni) are presented in Table 3, where it is shown the day post-treatment when a specific level of significance was reached. Significant differences were observed for the triple combination therapy from day 11 until the end of the study. Furthermore, a significant difference in average tumor volume and growth rate (Figure 6A and B respectively) was detected in animals subjected to combination therapy compared to those that received single drug-based treatments or drug vehicle when analyzed 30 days after the injection of MDA-MB-231 cells.

**Table 3:** Two-way ANOVA analysis of tumor growth inhibition by different treatments on MDA-MB-231 tumor xenografts.

Comparison		Significance Level, Days Post - treatment		
Treatment A	Treatment B	*P*<0.05	*P*<0.01	*P*<0.001
Control	Rosiglitazone	14	–	–
Control	Rosiglitazone	22	–	–
Control	Rosiglitazone	–	–	26
Control	Zileuton	26	–	–
Control	Ibuprofen	30	–	–
Control	Ros/Zil/Ibu	–	11	–
Control	Ros/Zil/Ibu	–	–	14
Control	Ros/Zil/Ibu	–	19	–
Control	Ros/Zil/Ibu	–	–	22
Control	Ros/Zil/Ibu	–	–	26
Control	Ros/Zil/Ibu	–	–	30
Rosiglitazone	Ros/Zil/Ibu	–	26	–
Rosiglitazone	Ros/Zil/Ibu	–	–	30
Rosiglitazone	Ibuprofen	26	–	–
Zileuton	Ros/Zil/Ibu	–	11	–
Zileuton	Ros/Zil/Ibu	14	–	–
Zileuton	Ros/Zil/Ibu	–	22	–
Zileuton	Ros/Zil/Ibu	–	–	26
Zileuton	Ros/Zil/Ibu	–	–	30
Ros/Zil/Ibu	Ibuprofen	–	14	–
Ros/Zil/Ibu	Ibuprofen	19	–	–
Ros/Zil/Ibu	Ibuprofen	–	22	–
Ros/Zil/Ibu	Ibuprofen	–	–	26
Ros/Zil/Ibu	Ibuprofen	–	–	30

GraphPad Prism Software was used to perform a two-way ANOVA of tumor growth data after treatment (data is graphed as tumor volume from day 11 to 30 post injection in figure 5A). Treatment A (first column) was compared to treatment B (second column) and the time it took (in days-post injection) to reach a significant level at *P*<0.05, *P*<0.01 or *P*<0.001 as indicated in columns 3, 4 and 5 respectively.

The tumors from mice treated with the combination treatment were clearly smaller than those from either the control group or individual drug treatment groups. Representative mice bearing a tumor from each treatment group are shown in Figure 5B.

Interestingly, the compounds assayed markedly reduced tumor volume and growth rate at concentrations that are ineffective when used alone. These results point to a synergistic effect that has the advantage of exposing mice to lower drug concentrations. This implies that ACSL4, LOX-5 and COX-2 interact functionally and represent an integrated system that operates in a concerted manner to regulate tumor growth and consequently the proliferation and metastatic potential of cancer cells.

## Discussion

This study was undertaken to confirm the effect of ACSL4 overxpression on tumor growth *in vivo*. We also aimed to determine whether a combinatorial inhibition of the ACSL4-LOX-5-COX-2 pathway affected tumor growth *in vivo*.

The first novel finding is that ACSL4 overexpression results in tumor development when injected into nude mice.

Our results show that ACSL4 overexpression, by itself, results in tumorigenic MCF-7 cells. Thus, ACSL4 overexpression is important in the promotion of the cell’s characteristics associated with cancer progression including increased proliferation and tumor growth promotion. Therefore, the *in vivo* xenograft model of breast cancer in which the expression of ACSL4 changes the cell’s potential for tumor formation, growth and development suggests that ACSL4 may be a novel therapy target.

With the use of this alternative xenogenic model, we further demonstrated that ACSL4 expression can be silenced in order to reduce the aggressiveness of the cell line and possibly the ability of cells to develop into mammary tumors. These results support our previous observations *in vitro* in which tetracycline treatment of MCF-7 Tet-Off/ACSL4 cells resulted in the inhibition of cell proliferation and migration [21].

Immunohistochemical analysis of tumor from MCF7-Tet-Off/ACSL4 breast cancer cells showed few positive cells expressing ER and very few stained for PR. These results suggest that the expression of ACSL4 negatively controlled the expression of ER and may be one of the first events in the transformation of the ER and PR positive phenotype into negative one as shown for the MDA-MD-231 human breast tumor xenograft.

These results concord with results showing that in samples from human breast tumor the expression of ACSL4 correlates with the absence of ER [18]. These results may also explain how inoculation of MCF-7 Tet-Off/ACSL4 human breast cancer cells in female mice results in the development of a tumor without requiring exogenously added estrogen.

Haughian J.M. et al, [33] described a very interesting study demonstrating that xenograft tumors comprised of ER^+^, PR^+^, T47D cells expressing a luminal gene signature produce tumors that contain a percentage of hormone resistant cells expressing a basal like triple negative signature. The tumor xenograft of T47D breast cancer cells were developed using ovariectomized nu/nu mice supplemented with silastic implants containing estradiol only or plus progestin MPA. The authors concluded that the outgrowth of the luminalbasal cell population is undesirable and demonstrates the necessity of using combination therapies that will target the hormone sensitive and insensitive cells. The implications of this data are grave for the development of resistance to ER-targeted endocrine therapies.

The xenograft model used in this paper is very different in many aspects to the T47D xenografts used in the Haughian paper. First of all, in our model we used a derivative of the MCF-7 breast cancer cell line (ER+ and PR+) that overexpresses ACSL4 in stable manner. This cell type was denominated MCF-7 Tet-Off/ACSL4. As a control we use the MCF-7-Tet-Off empty vector. Second, in both cases the animal used were normal nu/nu mice without hormone replacement. In the control experiments, out of ten animals injected with the MCF-7-Tett/off-empty vector cells, only two developed a very small tumor. On the other hand, the entire set of animals injected with the MCF-7 Tett/off-ACSL4 developed tumors. These tumors showed low levels of ER+ and PR+ cells. This tumor phenotype is a consequence of ACSL4 expression in the cells, since injection of MCF-7 control cells that do not overexpress ACSL4 resulted in no tumor formation. It is then possible that ACSL4-transformed MCF-7 cells show low levels of ER, as was shown in Figure 4, produce this tumor phenotype, but also that ACSL4 overexpression results in a more pronounced reduction in ER and PR receptor during the tumor growth. This suggestion is supported by the experiments showing that treatment of the animal inoculated with MCF-7 Tet-Off/ACSL4 breast cancer cells with doxycycline reduced the tumor volume and the remainder cells became positive for the expression of ER and PR. Therefore, the presence of a subpopulation of cells that express low levels of ER and PR as was the case for the T47D can be ruled out. If the MCF-7 Tet-Off empty vector would contain a subpopulation of cells with low levels of ER and PR they should form tumors in the absence of ACSL4 overexpression and they do not. These results strongly support the conclusion that the reduction in ER and PR receptor is due to ACSL4 overexpression and not to the presence of a subpopulation of cells expressing low levels of both receptors.

A high level of ACSL4 correlates with triple-negative breast cancers [18]. In fact, the MDA-MB-231 cell lines used in this study were triple negative cancer cells [24]. Notably, the combination of ACSL4, LOX-5 and COX-2 inhibitors effectively inhibited the growth of these cancer cells in mice. Thus, this combination therapy may provide an effective treatment option for triple-negative breast cancers since there are no specific treatment guidelines for triple-negative cancers, which appear to be very metastatic and have a poor prognosis [34].

Rosiglitazone as well as derivatives of troglitazone have been used either alone or in combination in experimental conditions to inhibit the growth of different tumor cell lines [35], [36]. Although the action of rosiglitazone has been attributed to its effects on PPARγ receptor, a very interesting report shows that rosiglitazone is working independently of its effects on PPARγ receptor in the inhibition of ovarian cancer cells [37]. This issue remains to be resolved [38].

Our results showed that rosiglitazone acts synergistically with inhibitors of LOX-5 and COX-2. A recent paper describes that rosiglitazone inhibited the induction of COX-2 in a human endothelial cell line [39]. These results agree with our previous demonstration that ACSL4 regulates the expression of COX-2 and with the present results showing the synergistic effect of ACSL4, LOX-5, and COX-2 inhibitors.

Altogether these results confirm the role of ACSL4 in tumor progression and suggest that the effect of rosiglitazone on tumor growth is due to its effects on ACSL4 activity as previously described [28].

In a recent paper [40], it is described that rosiglitazone inhibits cell proliferation and colony formation via PTEN expression-mediated apoptosis, in MCF-7 breast cancer cells. This growth inhibition was abolished by the use of a PPARγ antagonist or by PTEN knockdown by RNAi oligonucleotides. In the same paper the authors showed that rosiglitazone also inhibited cell proliferation in MDA-MB-231 breast cancer cells. However, no data showing the inhibition of colony formation or the effect of PPARγ antagonist or the PTEN RNAi oligonucleotide in growth inhibition is presented.

Rosiglitazone belongs to the thiazolodinedione family of drugs (TZDs) and is known to attenuate cell growth in carcinoma of various organs including breast, prostate, lung, colon, stomach, bladder and pancreas [41]. TZDs are a class of oral insulin-sensitizing agents, extensively used in the treatment of type 2 diabetes. The mechanism of TZD action is mediated largely through the activation of PPARγ, a member of the super family of ligand-activated nuclear transcription factors [42]: In the light of these cancer–specific effects, the potential use of these PPARγ agonists as chemopreventive agents has received much attention.

On the other hand, several lines of evidence have suggested that the inhibitory effect of TZDs on tumor proliferation is independent of PPARγ expression [43]. For example, the ability of TZD to inhibit cancer cell growth does not correlate with the levels of PPARγ expression, and there is a three orders of magnitude discrepancy between the concentration required to produce antitumor effects and that for PPARγ activation and also to modify insulin action [42], [43] Moreover it was possible to dissociate by structural modification the PPARγ activation and the antitumor effects in two prostate cancer cell lines [41].

TZDs may also exert non-genomic effects [41]. For example, rosiglitazone has been shown to activate 5′-AMP protein kinase through a PPARγ-independent mechanism [44]. In addition, in vitro studies performed with rat recombinant proteins have demonstrated that TZDs can directly inhibit the activity of one of the gene products of the acyl-CoA synthetases, the Acyl-CoA synthetase 4 (ACSL4) [45], [46].

The growth inhibition produced by TZDs was also linked to the arrest in G1 phase of the cell cycle, through the up-expression of the cyclin-dependent kinase inhibitors p^21^ and p^27^ [47] Despite these advances, the mechanism underling the antitumor effect of TZDs remains unclear. The PPARγ target genes that mediate the antiproliferative effects remain also elusive, as genomic responses to PPARγ activation in cancer cells are highly complicated [37], [41], [48], [49].

It seems that some of these targets for TZDs appear to be cell type specific due to the difference in signaling pathways regulating cell growth and survival in different systems. Therefore, it is not controversial that cells with different gene signature will use different signal transduction pathways to regulate cell growth, and that rosiglitazone may use two different pharmacological effects to inhibit cell growth in the MCF-7 and MDA-MB-231 cell lines. Moreover, two different TZDs may exert different effects in a single tumor cell. For example, troglitazone decreases cell growth of human C4-2 prostate cancer cells in vitro and in vivo by arresting cells in G(0)/G) phase of the cell cycle and induces apoptosis and decreased c-Myc protein. However rosiglitazone and pioglitazone do not reduce c-Myc protein in the same system [49]. Furthermore, the effect of troglitazone was not blocked by the PPARγ antagonist GW9662 and siRNA-mediated decreases in PPARγ protein. It seems that some of these targets for the different TZDs appear to be also cell type specific due to the difference in signaling pathways regulating cell growth and survival in different systems [49].

MCF-7 and the MDA-MB-231 breast cancer cells present different gene signatures. While MCF-7 cells are classified as luminal breast cancer expressing ER and PR, MDA-MB-231 correspond to the highly-aggressive basal-like “triple negative” cancer (ER and PR negative).

Our results show that rosiglitazone and troglitazone produced a synergistic inhibition of cell proliferation and migration when used in combination with lipooxigenase and ciclooxigenase inhibitors. The effectsof rosiglitazone and troglitazone are mimicked by Triacsin C, an inhibitor of ACSL4 activity not related to PPARγ activation (Table 1). We also show the same effect using the HS578T breast cancer cell line (Table 2). These results, together with our already published observations [21] showing that inhibition of ACSL4 expression in MDA-MB-231 transforms the cells into a non-aggressive phenotype, strongly suggests that the effect of rosiglitazone in MDA-MB-231 cell proliferation or the effect on tumor growth of rosiglitazone in combination with LOX and COX inhibitors results from ACSL4 inhibition and that the effect of rosiglitazone appears to be cell type-specific due to the difference in signaling pathways regulating cell growth and survival.

The increase in proliferation, invasion and migration is observed in cells overexpressing ACSL4 but not in cells exhibiting low levels of ACSL4. This specificity should increase the therapeutic index of combination-based therapies for ACSL4-overexpressing cancers. The *in vivo* xenograft model of breast cancer in which the expression of ACSL4 changes the cell’s potential for tumor formation, growth and development may be useful for testing novel targeted therapies.

COX-2 inhibitors significantly decrease breast tumor growth and clinical studies are underway to investigate their effect in patient populations. However in 2005 the New England Journal of Medicine published a review of COX-2 inhibitor cardiac safety which raised concerns over prolonged high-dose COX-2 inhibition [50].

The data presented here showing a synergistic inhibitory effect on tumor growth by ACSL4, LOX-5 and COX-2 inhibitors with the use of lower doses of the respective drugs may also result in the reduction of potentially severe side-effects. This model could also be useful to evaluate the preclinical safety and efficacy of novel adjuvant therapies for women with metastatic breast cancer.

## Materials and Methods

Dulbeccós modified Eagle medium (DMEM), penicillin-streptomycin solution trypsin-EDTA, G418 (Geneticin), and Opti-MEM were from GIBCO, Invitrogen Corporation (Grand Island, NY, USA). Fetal Calf Serum was from PAA laboratories GmbH (Pasching, Austria). Puromycin, tetracycline, doxycycline and 3-(4,5-dimethyl-2-thiazolyl)-2,5-diphenyl-2H-tetrazolium bromide (MTT) were purchased from Sigma Chemical Co. (St. Louis, MO, USA). Monoclonal mouse anti-Ki67, ERα and PR were from Upstate Group Inc. (Temecula, CA, USA). Santa Cruz Biothecnology, Inc. Horseradish peroxidase-conjugated goat_anti-rabbit and goat-anti-mouse secondary antibodies, polyvinylidene fluoride membrane was from Bio-Rad Laboratories (Hercules, CA, USA). Immun-Blot PVDF Membrane was from Bio-Rad Laboratories (Hercules, CA, USA). Enhanced chemiluminescence (ECL) was from GE Healthcare (Buckinghamshire, UK), Lipofectamine 2000 was from Invitrogen (Carlsbad, CA, USA). Tri reagent was from Molecular Research Center (Cincinnati, OH, USA).

### Cell Culture

Human breast cancer cell lines MDA-MB-231, MCF-7 and HS578T, were generously provided by Dr. Vasilios Papadoupoulus (Research Institute of the McGill University Health Centre, Montreal, Canadá) and obtained from the Lombardi Comprehensive Cancer Center (Georgetown University Medical Center, Washington D.C. USA). The cell lines were maintained in DMEM medium supplemented with 10% FBS plus 100 U/ml penicillin and 10 µg/ml streptomycin (complete DMEM).

### Stable Transfection of MCF-7 Tet-Off Cells with ACSL4 cDNA

The tetracycline-repressible MCF-7 cell line, designated MCF-7 Tet-Off, was used for stable transfection of ACSL4 cDNA under control of the tetracycline-response element using the Tet-Off Gene Expression System (Clontech laboratories, Inc, Mountain View, CA, USA,) as previously described [21]. After transfection, cells were maintained in complete DMEM supplemented with 0.3 µg/ml puromycin. After several weeks, colonies in which the ACSL4 expressed was regulated by tetracycline (2 µg/ml) were selected, cultured and subjected to different functional assays. Twenty clones of MCF-7 Tet-Off-induced repression of ACSL4, designated MCF-7 Tet-Off/ACSL4, were further maintained in complete DMEM. The MCF-7 Tet-Off/ACSL4 clones were analyzed to determine the expression of ACSL4 and COX-2 as well as the production of PGE_2_ as previously described [21]. Cells were also analyzed for cell proliferation, migration and invasion using the MTT, wound-healing and matrigel assays respectively, as previously described [21].

### Cell Proliferation Assay

Cell proliferation was measured by the MTT assay, as previously described [21], [51]. Cells were plated at a density of 4000 cells/well in 96-well plates with 10% FBS-supplemented D-MEM medium and allowed to adhere overnight at 37uC in a humidified, 5% CO2 atmosphere. The medium was then changed to serum-free medium. After 24 h, the cells were switched to 10% FBS-supplemented D-MEM medium and incubated for 96 h. Subsequently, MTT was added and incubated for 2:30 h at 37uC. Next, the formed formazan crystals were dissolved with DMSO. The absorbance 570 nm was determined using a Multi-detection microplate reader, Synergy HT, Biotek (Winooski, Vermont, USA).

### Wound-healing Assay

Cellular migration was measured by the wound healing assay, as previously described [21], [52]. Cells (7×10^5^ cells per well) were seeded in six-well plates. Cells were serum-starved for 24 h after which media was replaced (10% FBS medium) and the wound performed. Wound infliction was considered as 0 time and wound closure monitored for up to 24 h wound closure. Cell monolayer was wounding with a plastic tip across the monolayer cells. Wound closures were photographed by a phase contrast microscopy (40X) in the time point 12 h after scraping. The width of the wound was determined with the program Image Pro-Plus.

### Nude Mouse Xenograft Model

The experimental design followed a well-established female nude mouse model [53]. Cells (5×10^6^ cells) mixed with Matrigel Matrix (BD Biosciences) were injected into the right flank of female Foxn1 nu/nu Balb/c athymic nude mice, aged 6–8 weeks (UNLP Central Bioterium, Buenos Aires) and allowed to form tumors. Tumors were measured with callipers every other day (length and width) and the mice weighed. Mice were provided with free access to food, water and bedding at all time and were housed with a 12 h light/dark cycle in filter top cages containing a maximum of six mice per cage. Tumor volumes (mm^3^) were calculated by the formula: π/6×width^2^ (mm^2^)×length (mm) as described previously [53]. The experiment was terminated as previously described [54] in accordance with institutionally approved guidelines and tumors were harvested and fixed in 10% neutral buferred formalin and embedded in paraffin blocks for histological analysis and immunohistochemistry (IHC).

### Ethics Statement

This study was carried out in strict accordance with the recommendations in the Guide for the Care and Use of Laboratory Animals of the National Institutes of Health. The protocol was approved by the Institutional Ethical Commitee from the School of Medicine, University of Buenos Aires (ID:093/10 CD, Shool of Medicine).

### *In vivo* Therapy of Solid Tumors in Mice

For MDA-MB-231 tumor xenografts, pathogen-free female athymic nu/nu (nude) mice of 6–8 weeks of age were used. Nude mice were subcutaneously injected with 5×10^6^ MDA-MB-231 human breast cancer cells mixed with Matrigel Matrix (BD Biosciences) on the right flank. Four days after cell injection the tumor-bearing mice were randomized into the following five groups (five animals per group) and the animals received intraperitoneal injections for 25 consecutive days with:

The dose of the inhibitors used was calculated taken into account the minimal dose of each individual inhibitor that produce a significant effect in the xenograft MDA-MB-231 model. Unitary doses were: rosiglitazone (2.4 mg/day); zileuton (0.50 mg/day); ibuprofen (2.5 mg/day). Doses employed for the combinatorial therapy were: rosiglitazone (0.6 mg/day); zileuton (0.24 mg/day); ibuprofen (1.2 mg/day). In all cases, drugs were administered once a day by ip injection during 26 days.

Group 1 (MDA-MB-231 cell xenografts treated with vehicle), Group 2 ((MDA-MB-231 cell xenografts treated with rosiglitazone, Group 3 (MDA-MB- 231 cell xenografts treated with zileuton, Group 4 (MDA-MB-231 cell xenografts treated with ibuprofen and Group 5 (MDA-MB-231 cell xenografts treated with a combination of the three drugs at the same doses used for the individual injections).

Animals were maintained in pathogen-free conditions and procedures were performed in accordance with recommendations for the proper use and care of laboratory animals. Tumors were measured as described above. Individual animal weights were recorded before and after treatment.

### Histological Analysis

Tumors were collected, immediately fixed in 10% formalin (pH 7), paraffin-embedded and sectioned into 5 µm for histochemical analysis. Subsequently, the dewaxed and alcohol-hydrated sections were stained with hematoxylin–eosin, dehydrated and mounted in Histomount (Zymed Laboratories, San Francisco, USA). Finally, stained sections were carefully examined by a pathologist and photos of from one representative animal per group are shown.

### Immunohistochemistry

Three micron sections of the paraffin blocks were cut onto APES coated slides. The slides were dewaxed in xylene and rehydrated through graded alcohols to phosphate saline buffer. Specimens were stained for detection of the reference breast cancer antigens: ERα, PR, Human Epidermal Growth Factor Receptor-2 (HER2), and Ki-67 (Proliferation marker).

All incubations were carried out at room temperature. The antigen was retrieved by immersing slides in citrate buffer (pH 6) in a pressure cooker for 120 sec. After the incubation with the specific antibodies, the stained procedure was performed using the Immpress Universal reagent anti-mouse/rabbit Ig from Vector laboratories according with the manufacter intructions.

### RNA Extraction and Semiquantitative RT-PCR

Total RNA from MCF-7 cells subjected to different transfection protocols was extracted using Tri reagent (Molecular Research Center) following the manufacturer’s instructions. Primers used for ERα amplification (amplicon size 750 bp) were: sense primer, 5′-AACACAAGCGCCAGAGAGAT-3′ and the antisense primer, 5′-GATGTGGGAGAGGATGAGGA- 3′. Primers used for PR amplification (amplicon size 576 bp) were: sense primer, 5′-AAATCATTGCCAGGTTTTCG-3′ and the antisense primer, 5′-TCACCATTCCTGCCAATATC- 3′. The amplified L19 ribosomal protein product of each sample (amplicon size 500 bp) was used as housekeeping gene [55]. Specific primers for human L19 were: sense: 5′-AGTATGCTCAGGCTTCAGAA- 3′, and antisense: 5′-TTCCTTGGTCTTAGACCTGC- 3′. The reaction conditions were one cycle of 94°C for 5 min, followed by 32 cycles for ERα or 25 for L19 of 94°C for 30 sec, 60°C for 30 sec, 72°C for 45 sec, and finally one cycle of 72°C for 10 min. The number of cycles used was optimized for each gene to fall within the linear range of PCR amplification. PCR products were resolved on 1.5% (wt/vol) agarose gel containing ethidium bromide. Gel images were digitally recorded and amplicon levels were quantified by the computer-assisted image analyzer Gel-Pro (IPS, North Reading, MA, USA).

### Western Blot

Cell cultures were washed with PBS, scraped in 10 mM Tris-HCl (pH 7.4), 250 mM sucrose, 0.1 mM EDTA, 10 µM leupeptin, 1 µM pepstatin A, and 1 mM EGTA, homogenized with a Pellet pestle motor homogenizer (Kimble Kontes, Vineland, NJ), and centrifuged at 100×g for 5 min. Total lysis proteins (60 µg) were separated on SDS-PAGE and electro-transferred to poly (vinylidene difluoride) membranes (Bio-Rad Laboratories) as described previously [21]. Membranes were then incubated with 5% fat-free powdered milk in 500 mM NaCl, 20 mM Tris-HCl (pH 7.5), and 0.5% Tween 20 for 60 min at room temperature, with gentle shaking. The membranes were then rinsed twice in 500 mM NaCl, 20 mM Tris-HCl (pH 7.5), and 0.5% Tween 20 and incubated overnight with the appropriate dilutions of primary antibody at 4 oC: 1∶300 rabbit polyclonal anti-P4, 1∶400 mouse monoclonal anti-ER 1∶5,000 mouse monoclonal anti-tubulin. Bound antibodies were developed by incubation with secondary antibody 1∶5,000 goat anti-rabbit and 1∶5,000 goat anti-mouse horseradish peroxidase conjugated and detected by chemiluminescence. The inmunoblots were then quantitated using Gel Pro Analyzer.

### Statistical Analysis

Data analysis was performed using GraphPad Prism Software 5.01 (La Jolla, CA, USA). Tumor growth and tumor response to treatment was compare using two-way ANOVA, post test Bonferroni. Tumor growth rate was analyzed by one-way ANOVA. *P*<0.05 was consider statistically significant.
